# Repression of *FLOWERING LOCUS C* and *FLOWERING LOCUS T* by the *Arabidopsis* Polycomb Repressive Complex 2 Components

**DOI:** 10.1371/journal.pone.0003404

**Published:** 2008-10-14

**Authors:** Danhua Jiang, Yuqi Wang, Yizhong Wang, Yuehui He

**Affiliations:** 1 Department of Biological Sciences, National University of Singapore, Singapore, Republic of Singapore; 2 Temasek Life Sciences Laboratory, Singapore, Republic of Singapore; University of California Davis, United States of America

## Abstract

Polycomb group (PcG) proteins are evolutionarily conserved in animals and plants, and play critical roles in the regulation of developmental gene expression. Here we show that the *Arabidopsis* Polycomb repressive complex 2 (PRC2) subunits CURLY LEAF (CLF), EMBRYONIC FLOWER 2 (EMF2) and FERTILIZATION INDEPENDENT ENDOSPERM (FIE) repress the expression of *FLOWERING LOCUS C* (*FLC*), a central repressor of the floral transition in *Arabidopsis* and *FLC* relatives. In addition, CLF directly interacts with and mediates the deposition of repressive histone H3 lysine 27 trimethylation (H3K27me3) into *FLC* and *FLC* relatives, which suppresses active histone H3 lysine 4 trimethylation (H3K4me3) in these loci. Furthermore, we show that during vegetative development *CLF* and *FIE* strongly repress the expression of *FLOWERING LOCUS T* (*FT*), a key flowering-time integrator, and that CLF also directly interacts with and mediates the deposition of H3K27me3 into *FT* chromatin. Our results suggest that PRC2-like complexes containing CLF, EMF2 and FIE, directly interact with and deposit into *FT*, *FLC* and *FLC* relatives repressive trimethyl H3K27 leading to the suppression of active H3K4me3 in these loci, and thus repress the expression of these flowering genes. Given the central roles of *FLC* and *FT* in flowering-time regulation in *Arabidopsis*, these findings suggest that the CLF-containing PRC2-like complexes play a significant role in control of flowering in *Arabidopsis*.

## Introduction

The transition from a vegetative to a reproductive phase (i.e., flowering) is a major developmental switch in the plant life cycle that must be properly timed to ensure maximal reproductive success. In *Arabidopsis thaliana*, this transition is genetically controlled by several pathways, including the autonomous pathway, the photoperiod pathway and the vernalization pathway, which form a regulatory network [1], [2]. This network integrates the endogenous developmental state of the plant with environmental cues (e.g., day length and temperature) to precisely control the timing of the floral transition [1], [2].

A key component in this regulatory network in *Arabidopsis* is FLC, a MADS box transcription factor that quantitatively inhibits the floral transition [3], [4]. *FLC* expression is delicately controlled by various activators and repressors. The autonomous pathway, which includes *FVE* [5], [6], *FCA* [7] and *FLOWERING LOCUS D* (*FLD*) [8], constitutively represses *FLC* expression to promote flowering, whereas *FRIGIDA* (*FRI*) activates *FLC* expression to delay flowering [9]. The vernalization pathway also represses *FLC* expression in response to a prolonged cold exposure (a typical winter) to accelerate flowering in *Arabidopsis* [10], [11]. Besides *FLC*, in the *Arabidopsis* genome there are five close *FLC* relatives including *FLOWERING LOCUS M* (*FLM*), *MADS AFFECTING FLOWERING 2* (*MAF2*), *MADS AFFECTING FLOWERING 3* (*MAF3*), *MADS AFFECTING FLOWERING 4* (*MAF4*) and *MADS AFFECTING FLOWERING 5* (*MAF5*); these *FLC* relatives also appear to repress the floral transition [12], [13].

Chromatin modification plays an important role in the regulation of *FLC* expression. Activation of *FLC* expression in the presence of *FRI* is associated with the H3K4 trimethylation and also requires deposition of the histone variant H2A.Z in *FLC* chromatin [14], [15], [16]. The autonomous-pathway represses *FLC* expression partly through generating repressive histone modifications in *FLC* chromatin. *FLD* is involved in the H3K4 demethylation and deacetylation of *FLC* chromatin [8], [17], [18]; *FCA* functions closely with *FLD* and is involved in H3K4 demethylation in *FLC* chromatin [18]; *FVE* is partly involved in the histone deacetylation of *FLC* chromatin [5], [8]. In addition, histone H4 dimethylation at arginine 3 (H4R3) in *FLC* chromatin by Type I and Type II arginine methyltransferases is also associated with *FLC* repression [19], [20], [21]. Furthermore, small RNA-mediated repressive histone modifications are also linked to *FLC* repression [22], [23]. Recent studies also reveal that vernalization leads to repressive histone modifications in *FLC* chromatin such as increased trimethylation of histone H3 at lysine 9 and H3K27, and H4R3 dimethylation [24], [25], [26], [27].

*FLC* inhibits the floral transition partly by reducing expression of a key flowering-time integrator, *FT* [28]. *FT* was first identified as a component of the photoperiod pathway, which promotes flowering in response to increased day length [29], [30], [31]. In the presence of light, *FT* expression is activated by *CONSTANS* (*CO*), another component in the photoperiod pathway [31]. *FT* is expressed in the vasculature [32], and subsequently, FT proteins are translocated from veins to the shoot apex to promote flowering [33], [34], [35]. FLC binds to the *FT* locus and represses its expression, and thus antagonizes the activation by CO [28]. Hence, *FT* acts as a flowering-time integrator that integrates signals from the photoperiod pathway and the *FLC*-mediated flowering pathways to promote the *Arabidopsis* flowering. Recent studies indicate that chromatin modification may play a role in the regulation of *FT* expression. It has been shown that LIKE HETEROCHROMATIN PROTEIN 1 (LHP1) directly interacts with *FT* chromatin and represses *FT* expression [36], [37], [38]; in addition, recent whole-genome analysis of H3K27 trimethylation in *Arabidopsis* has revealed that this repressive mark is associated with *FT* chromatin [39]. However, how H3K27me3 is deposited in *FT* chromatin and its role in *FT* regulation remain elusive.

Repressive H3K27me3 is deposited by the PRC2 complex in *Drosophila*. PRC2 is composed of four core proteins including Enhancer of zeste (E(z); an H3K27 methyltransferase), Extra sex comb (Esc), Suppressor of zeste 12 (Su(z)12) and p55, and deposits trimethyl H3K27 to silence the expression of homeotic genes in *Drosophila* (reviewed in [40]). Homologs of *Drosophila* PRC2 components have also been identified in *Arabidopsis*, and play important roles in the control of plant developmental processes such as floral induction, flower organogenesis, seed development and sporophyte development (reviewed in [41], [42]). To date, a PRC2-like complex composed of MEDEA (MEA), FIE, FERTILIZATION INDEPENDENT SEED 2 and MULTICOPY SUPPRESSOR OF IRA1 (MSI1), which are relatives of E(z), Esc, Su(z)12 and p55 respectively, has been biochemically characterized [43], [44]. This complex represses the MADS box gene *PHERES1* during seed development and thus controls this developmental process [45], [46].

Recent studies have also shown that CLF, an *Arabidopsis* homolog of E(z), directly mediates the repression of *AGAMOUS* (*AG*) via H3K27 trimethylation and thus controls floral organogenesis [47], [48]. *CLF* plays multiple roles in plant development, and also directly represses the expression of *SHOOTMERISTEMLESS* (*STM*) and a flowering gene, *AGAMOUS LIKE 19* (*AGL19*), during vegetative development [48], [49]. Recent studies also reveal that VERNALIZATION 2 (VRN2), a homolog of Su(z)12, plays an important role in the vernalization-mediated *FLC* repression [50]. *VRN2* is required for *FLC* repression by vernalization treatment [50]; VRN2 forms a complex with CLF, SWINGER (SWN; another homolog of E(z)), FIE and VERNALIZATION INSENSITIVE 3 to repress *FLC* expression in response to vernalization treatment [51]. In addition, *EMF2*, a relative of *VRN2* and *Su(z)12*, also plays an important role in sporophyte development, and maintains vegetative development by repressing the floral induction [52], [53], [54]. However, the underlying mechanisms of the *EMF2*-mediated floral repression are unclear [54].

Here we report that *Arabidopsis* PRC2-like complex subunits CLF, EMF2 and FIE repress the expression of *FLC* and *FLC* relatives including *MAF4* and *MAF5*, and that CLF directly binds to and mediates the deposition of H3K27me3 in *FLC*, *MAF4* and *MAF5* chromatin. Furthermore, we show that during vegetative development CLF and FIE strongly repress *FT* expression, and that CLF also directly interacts with and mediates the deposition of H3K27me3 in *FT* chromatin. Theses results imply that PRC2-like complexes containing CLF, EMF2 and FIE deposit repressive H3K27me3 in and directly repress the expression of these flowering genes, and thus control the flowering program in *Arabidopsis*.

## Results

### PRC2 Subunits CLF, EMF2 and FIE Repress the Expression of *FLC*, *MAF4* and *MAF5* in Vegetative Development

**Figure 1:**
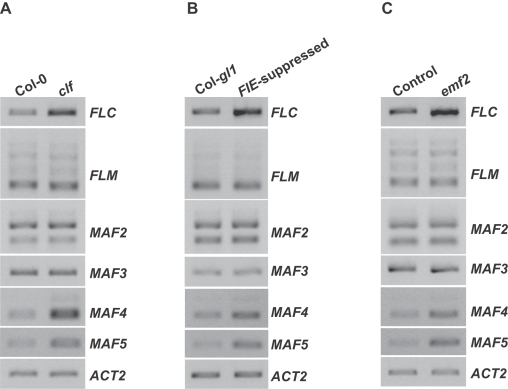
PRC2 subunits CLF, EMF2 and FIE repress the expression of *FLC* and *FLC* relatives. (A) Analysis of the expression of *FLC* and *FLC* relatives in *clf* seedlings by RT-PCR. *ACTIN2* (*ACT2*) served as an internal control. (B) Analysis of the expression of *FLC* and *FLC* relatives in seedlings of Col-*gl1* in which *FIE* is co-suppressed [55]. (C) Analysis of the expression of *FLC* and *FLC* relatives in *emf2* seedlings. *emf2* homozygotes were isolated from a selfed population of an *emf2* heterozygote. “Control” is a mixture of wild-type like seedlings consisting of Col and *emf2* heterozyges isolated from the same population as *emf2* homozygotes.

Arabidopsis PRC2-like complex components including VRN2, FIE, SWN and CLF are required for the vernalization-mediated *FLC* repression [50], [51]. We sought to investigate PRC2-mediated *FLC* repression in *Arabidopsis* plants grown in normal conditions (i.e., without vernalization treatment). In addition, the expression of *FLC* relatives such as *FLM*, *MAF4* and *MAF5*, like *FLC* expression, is also regulated by chromatin modification [14], [15]; hence, it was also of interest to investigate whether PRC2-like complexes repress the expression of *FLC* relatives. First, we addressed the role of *CLF* in the regulation of *FLC* and *FLC* relatives. Transcript levels of these genes were examined in seedlings of the *clf-81* mutant carrying a lesion in the SET domain of CLF [48]. We found that *FLC*, *MAF4* and *MAF5* were de-repressed in *clf*, whereas transcripts of *FLM*, *MAF2* and *MAF3* in *clf* remained at levels similar to wild-type Col (Figure 1A); hence, *CLF* plays an essential role in repressing the expression of *FLC*, *MAF4* and *MAF5* during vegetative development. Secondly, we investigated the role of *FIE* in the regulation of *FLC* and *FLC* relatives using *FIE*-suppressed seedlings [55] (note that *fie* alleles can not be transmitted through the female gamete [56]). Consistent with a recent report [51], in *FIE*-suppressed seedlings *FLC* expression was de-repressed (Figure 1B); furthermore, we found that *MAF4* and *MAF5* were also de-repressed, whereas *FLM*, *MAF2* and *MAF3* in these seedlings were expressed at levels similar to those in the wild type (Figure 1B). Hence, like *CLF*, *FIE* also selectively represses the expression of *FLC*, *MAF4* and *MAF5*.

CLF has been shown to directly interact with EMF2 and these two proteins may be part of a PRC2-like complex involved in the regulation of vegetative development in *Arabidopsis* [57]. We therefore examined transcript levels of *FLC* and *FLC* relatives in *emf2* seedlings. Indeed, *FLC*, *MAF4* and *MAF5*, but not *FLM*, *MAF2* or *MAF3*, were de-repressed in *emf2* (Figure 1C). Hence, like *CLF* and *FIE*, *EMF2* also selectively represses *FLC*, *MAF4* and *MAF5* expression during vegetative development. Together, these data suggest that there is a CLF-containing PRC2-like complex composed of at least EMF2 and FIE, which acts to repress *FLC*, *MAF4* and *MAF5* expression during vegetative development.

### *CLF* and *FIE* also Repress *FT* Expression in Vegetative Development

**Figure 2:**
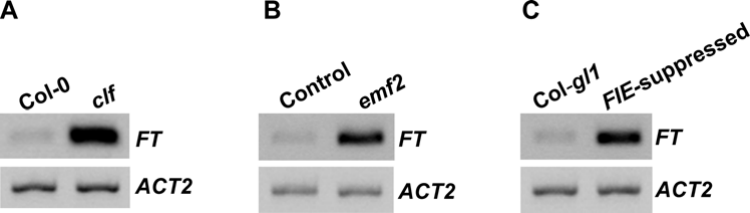
PRC2 subunits CLF, EMF2 and FIE repress *FT* expression. (A) Analysis of *FT* expression in *clf* seedlings by RT-PCR. *ACT2* served as an internal control. (B) Analysis of *FT* expression in *emf2* seedlings. The control is as described in Figure 1C. (C) Analysis of *FT* expression in seedlings of Col-*gl1* in which *FIE* is co-suppressed.

The de-repression of *FLC* and *MAF*s in *clf*, *emf2* and *FIE*-suppressed plants was expected to lead to late flowering because the elevated expression of these genes alone causes late flowering [3], [4], [13]; however, these mutant plants all are early-flowering [47], [52], [55]. These early-flowering phenotypes are likely due to increased or ectopic expression of genes that promote flowering. *CLF* and *EMF2* have been shown to repress the expression of the flowering promoter *AGL19* [49]; furthermore, ectopic expression of *AG* in *clf* and *emf2* may also partly contribute to the early-flowering phenotypes [47], [54]. In addition, a very recent report shows that *FT* expression is upregulated in 21-day-old *clf* mutant plants grown under continuous light [58], indicating that *FT* de-repression may partly account for the early-flowering phenotype of *clf*. We examined *FT* mRNA levels in young Col and *clf* seedlings to address whether *FT* is also de-repressed in *clf* mutants before the floral transition. Indeed, *FT* expression was greatly de-repressed in *clf* seedlings (Figure 2A). These data together with recent findings [58] suggest that *CLF* represses *FT* expression throughout vegetative development.

Recently, it has been shown that *FT* mRNA levels are higher in *emf2* relative to Col [54], [58], but the role of *EMF2* in *FT* repression is unclear [54]. We also examined *FT* mRNA levels in *emf2* seedlings. Consistent with the recent reports [54], [58], *FT* expression was de-repressed in *emf2* (Figure 2B). Because FIE may be part of the PRC2-like complexes containing EMF2 and CLF [41], we examined *FT* transcript levels in *FIE*-suppressed seedlings to determine whether *FIE* is also involved in *FT* repression, and found that *FT* is strongly de-repressed in these seedlings compared to the control Col-*gl1* seedlings (Figure 2C). Taken together, these data suggest that a PRC2-like complex containing CLF, EMF2 and FIE, represses *FT* expression in vegetative development to repress the floral transition.

**Figure 3:**
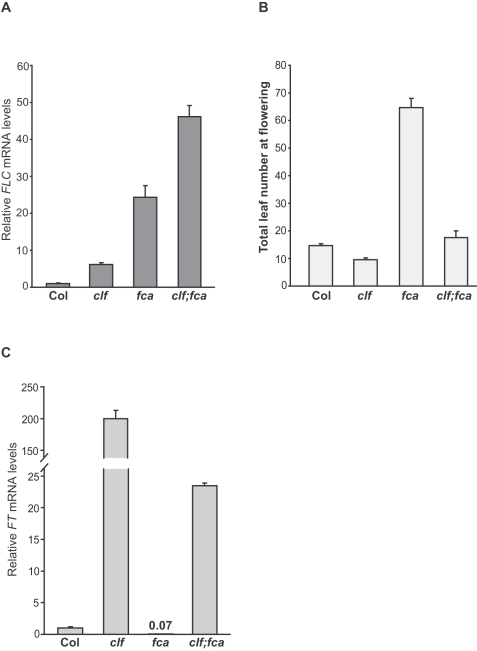
The genetic interaction of *clf* with *fca*. (A) Relative *FLC* mRNA levels in seedlings of *clf*, *fca* and *clf*;*fca* quantified by real-time PCR. Bars represent mean values±SD. (B) Flowering times of *clf*, *fca* and *clf*;*fca* mutants grown in long days. The total number of primary rosette and cauline leaves at flowering was scored, and for each line at least 10 plants were scored. The values shown are means±SD. (C). Relative *FT* mRNA levels in seedlings of *clf*, *fca* and *clf*;*fca* quantified by real-time PCR. Bars represent mean values±SD.

Interestingly, although these PRC2 subunits repress both *FLC* and *FT* expression and FLC directly represses *FT* expression, loss or suppression of the functions of these subunits leads to a greater *FT* derepression compared to *FLC* derepression (Figure 1 and Figure 2; also refer to Figure 3), suggesting that PRC2-like complexes have a repressive effect on *FT* expression much stronger than that on *FLC* expression.

### *CLF* Acts in Partial Redundancy with Part of the Autonomous Pathway to Repress *FLC* Expression in the Absence of Vernalization

The autonomous pathway constitutively represses *FLC* expression to promote flowering, and part of this pathway is involved in the generation of repressive histone modifications in *FLC* chromatin [59]. The autonomous-pathway repressor *FCA* directly binds to the *FLC* locus and is involved in the H3K4 demethylation of *FLC* chromatin [18]. Recent studies in mouse embryonic stem cells have suggested the coordinated regulation of H3K4 demethylation and PRC2-mediated repressive histone modifications in maintaining transcriptional gene repression [60]. Hence, it was of interest to examine the genetic interaction of *clf* with *fca*. We introduced *clf* into the *fca* mutant, and quantified *FLC* transcripts in *clf*, *fca* and *clf;fca* seedlings by real-time quantitative PCR. Consistent with previous findings [7], *FLC* was highly expressed in *fca* mutants (Figure 3A); however, *FLC* was further de-repressed in *clf;fca* and *FLC* mRNA levels in the double mutants were much higher than those in *fca* or *clf* (Figure 3A). Hence, *CLF* acts in partial redundancy with *FCA* to repress *FLC* expression in the absence of vernalization.

We further measured flowering times of *fca* and *clf;fca* mutants grown in long days. Although *FLC* was so highly expressed in *clf;fca*, the double mutants flowered much earlier than *fca* (Figure 3B). As noted above, *FT* is de-repressed in *clf*; hence, it is likely that the early-flowering phenotype of *clf;fca* is partly due to *FT* derepression. We quantified *FT* transcript levels in *clf*, *fca* and *clf;fca* seedlings. *FT* mRNA levels increased about 200 fold in *clf* relative to Col, whereas *FT* expression was suppressed in *fca* because of elevated *FLC* expression (Figure 3C). Furthermore, *FT* expression was partially suppressed in *clf;fca*, but *FT* transcript levels in the double mutant were still higher than those in *fca* (Figure 3C), suggesting that the early-flowering phenotype of *clf;fca* is at least partly due to the elevated *FT* expression.

### CLF Directly Interacts with the *FLC*, *MAF4*, *MAF5* and *FT* Chromatin

**Figure 4:**
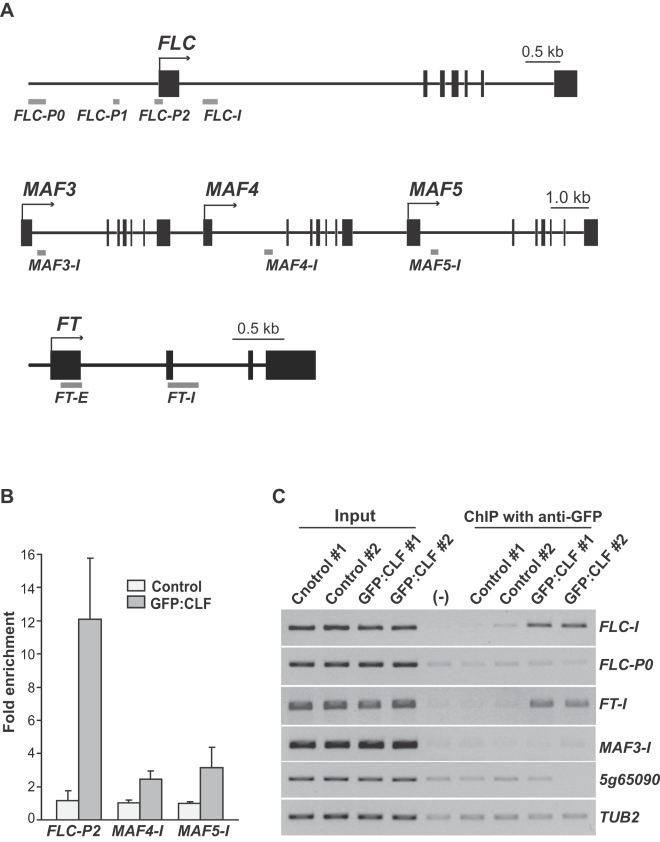
CLF binds to the *FLC*, *MAF4*, *MAF5* and *FT* loci. (A) Genomic structures of *FLC*, *FT* and the gene cluster of *MAF3*, *MAF4* and *MAF5* and the regions examined after ChIP. The transcription start sites are indicated by arrows; black boxes represent exons. (B) Binding of CLF to *FLC*, *MAF4* and *MAF5* chromatin. DNA fragments of *FLC-P2*, *MAF4-I* and *MAF5-I*, immunoprecipitated with anti-GFP from seedlings of a *35S:GFP:CLF clf* transgenic line (Ws background) and Ws (with native CLF; served as control), were quantified by real-time quantitative PCR and subsequently normalized to an internal control (*TUBLIN 2*; *TUB2*). The fold enrichments of the *35S:GFP:CLF clf* line over the control (Ws) are shown, and the values shown are means±SD. (C) Binding of CLF to *FT* and *FLC* chromatin analyzed by ChIP-PCR. Two independent immunoprecipitations were shown. “Input” is the total DNA prior to immunoprecipitation (diluted 640 times); “(-)” is the negative control for immunoprecipitation, residual DNA from the rabbit IgG immunoprecipitation. The constitutively expressed *TUB2*, a nontarget gene of CLF, was used as an internal control for PCR.

As noted above, CLF, EMF2 and FIE repress *FLC*, *MAF4*, *MAF5* and *FT* expression, however, it was not known whether these PRC2 subunits acted directly on these genes or indirectly. Using chromatin immunoprecipitation (ChIP), we first examined whether CLF directly interacts with the *FLC*, *MAF4*, and *MAF5* loci. Specifically, genomic DNA was immunoprecipitated using an antibody recognizing GFP from seedlings of a *35S:GFP:CLF clf* transgenic line in which GFP:CLF fully functions and its distribution mimics that of the endogenous CLF [48], and subsequently, the genomic DNA was quantified by real-time PCR or examined by PCR if the amounts of DNA in a ChIP sample were too low to be quantified. We found that both the region (*FLC-P2*) around the transcription start site (TSS) and 5′ part of Intron I of *FLC* (*FLC-I*) were greatly enriched, whereas a 5′ promoter region 1.8 kb upstream from the TSS in *FLC* was not enriched (Figure 4B and 4C). Moreover, we found that regions in the first introns of *MAF4* and *MAF5* were also enriched (Figure 4B), whereas *MAF3*, a close relative of *MAF4* and *MAF5* located immediately upstream *MAF4* (Figure 4A), and *At5g65090*, the gene immediately downstream *MAF5* (*At5g65080*), were not enriched (Figure 4C). Together, these data suggest that CLF selectively binds to *FLC*, *MAF4* and *MAF5 in vivo* to repress the expression of these genes.

To examine whether CLF directly interacts with the *FT* locus, using ChIP-PCR we checked the middle region of *FT* (*FT-I*; see Figure 4A), a region where FLC has been shown to bind [28]. As shown in Figure 4C, *FT* fragments were strongly enriched in the ChIP samples from the *35S:GFP:CLF clf* transgenic line. Hence, CLF directly interacts with *FT* chromatin to represses *FT* expression during vegetative development.

### Loss of CLF Function Leads to Reduction in Global H3K27 Trimethylation, but not in H3K27 Dimethylation during Vegetative Development

**Figure 5:**
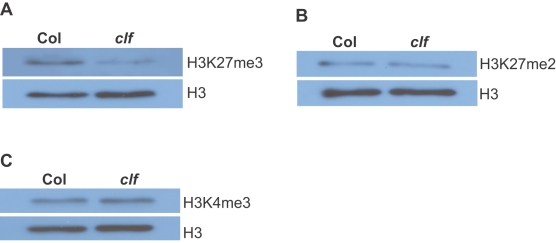
Analysis of histone methylation in the *clf* mutant by immunoblotting. (A) Analysis of H3K27me3 in Col and *clf* seedlings. Histone extracts from Col and *clf* were blotted with anti-trimethyl H3K27 (top panel) and anti-H3 (bottom panel). (B) Analysis of H3K27me2 in Col and *clf* seedlings. Histone extracts were blotted with anti-dimethyl H3K27 (top panel). (C) Analysis of H3K4me3 in Col and *clf* seedlings. Histone extracts were blotted with anti-trimethyl H3K4 (top panel).

CLF is a plant homolog of the *Drosophila* E(z), an H3K27 methyltransferase in the Esc-E(z) PRC2 complex [61], [62]. Previous studies have shown that E(z) and E(z)H2, the mammalian homolog of E(z), display PRC2-complex-dependent H3K27 methyltransferase activities on chromatin substrate (reviewed in [63]). It has been shown that CLF is partly required for H3K27me3 in CLF-target genes such as *AG* and *STM* [48]. We compared global histone methylation levels in *clf* and wild-type Col seedlings, including H3K27 dimethylation, H3K27 trimethylation and H3K4 trimethylation. Levels of trimethyl H3K27 were strongly reduced in *clf* relative to Col (Figure 5A), whereas levels of dimethyl H3K27 and trimethyl H3K4 in *clf* were similar to those in Col (Figure 5B and 5C), indicating that CLF is likely to be a histone methyltransferase catalyzing H3K27 trimethylation. Interestingly, lower levels of trimethyl H3K27 were still detected in *clf* mutant seedlings, which may be deposited by PRC2-like complexes containing CLF relatives including SWN and MEA.

### CLF Mediates the Deposition of H3K27me3 in *FLC*, *MAF4*, *MAF5* and *FT*

**Figure 6:**
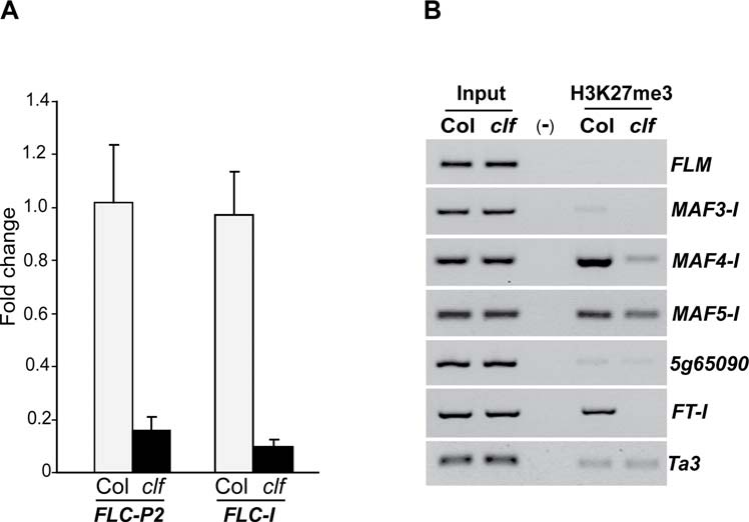
CLF mediates the deposition of H3K27me3 in the *FLC*, *MAF4*, *MAF5* and *FT* chromatin. (A) Levels of trimethyl H3K27 in *FLC* chromatin in Col and *clf* seedlings determined by real-time quantitative PCR. Amounts of DNA fragments after ChIP were quantified and subsequently normalized to an internal control (*TUBULIN 8*). The fold changes of *clf* over Col are shown, and the values shown are means±SD. Examined regions are as illustrated in Figure 4A. (B) H3K27 trimethylation state in *FLC* relatives and *FT* in Col and *clf* seedlings analyzed by ChIP-PCR. “(-)” is the negative control (without antibody) for immunoprecipitation. *Ta3* served as an internal standard for the ChIP-PCR indicating that the amount of total immunoprecitated DNA from *clf* is similar to that from Col. Representative ChIP-PCR results are shown in the gel picture.

As noted above, CLF mediates global H3K27 trimethylation during vegetative development; in addition, recent whole-genome analysis of H3K27 trimethylation in *Arabidopsis* has revealed that this modification is associated with *FLC* chromatin in the absence of vernalization treatment [39], which is likely deposited by a CLF-containing PRC2-like complex. It was of interest to examine the H3K27 trimethylation state in *FLC*, *MAF4* and *MAF5* in *clf* seedlings. As shown in Figure 6A, H3K27me3 was enriched in the promoter region *FLC-P2* and 5′ part of Intron I of *FLC* (*FLC-I*) in Col and loss of CLF activities significantly reduced the levels of trimethyl H3K27, consistent with the derepression of *FLC* in *clf* (Figure 3A). Furthermore, H3K27me3 was also enriched in *MAF4* and *MAF5* in the wild type and strongly reduced in *clf* (Figure 6B). In contrast, very little trimethyl H3K27 was detected in the neighboring genes including *MAF3* and *At5g65090* (Figure 6B). In addition, we did not detect trimethyl H3K27 in *FLM* (Figure 6B), another close relative of *FLC* and *MAF*s. Together, these data show that CLF mediates the deposition of trimethyl H3K27 selectively in *FLC*, *MAF4* and *MAF5*, consistent with the selective de-repression of these three genes, but not *FLM* or *MAF3* in *clf*.

We also found that H3K27me3 was enriched in *FT* chromatin in Col as reported previously [39], and that H3K27me3 in *FT* was nearly eliminated in *clf* (Figure 6B), consistent with the drastic de-repression of *FT* in *clf* (Figure 3C). As described above, CLF, EMF2 and FIE may be part of a PRC2-like complex that represses *FT* expression. Together, these data suggest that a CLF-containing PRC2-like complex may be responsible for depositing repressive H3K27me3 in *FT* chromatin.

### CLF-Dependent H3K27 Trimethylation Suppresses H3K4 Trimethylation in its Target-Gene Chromatin

**Figure 7:**
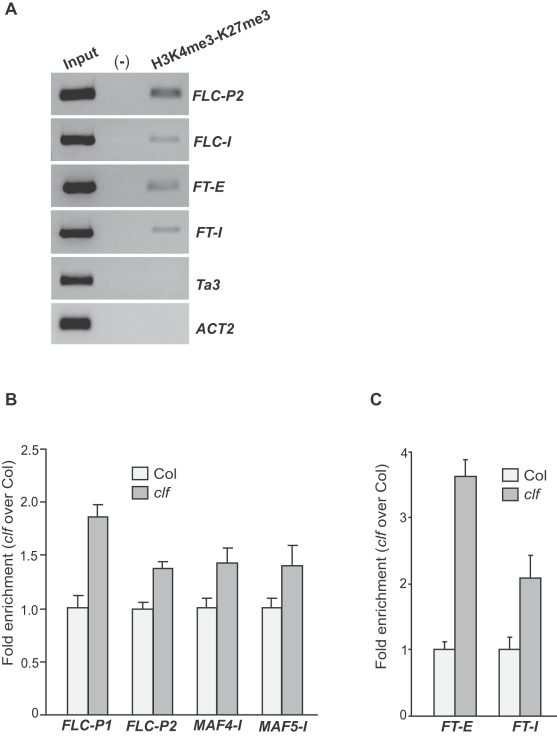
Interaction of the CLF-dependent H3K27 trimethylation with H3K4 trimethylation in its target-gene chromatin. (A) Sequential ChIP analysis of *FLC* and *FT* chromatin. The chromatin from wild-type Ws seedlings was immunoprecipitated first with anti-trimethyl H3K4 and second with anti-trimethyl H3K27. Examined regions are as illustrated in Figure 4A. “Input” is the total DNA prior to the first immunoprecipitation (diluted 800 times); *Ta3*, a heterochromatic locus lacking of H3K4me3 and *ACT2*, a constitutively expressed locus lacking of H3K27me3, served as negative controls. “(-)” is the negative control for immunoprecipitation, residual DNA from the rabbit IgG immunoprecipitation. (B) Levels of trimethyl H3K4 in the *FLC*, *MAF4* and *MAF5* chromatin in *clf* seedlings relative to Col determined by real-time quantitative PCR. Amounts of DNA fragments from Col and *clf* seedlings after ChIP were quantified and subsequently normalized to an internal control (*TUB2*). The fold enrichments of *clf* over Col are shown, and the values shown are means±SD. (C) Levels of trimethyl H3K4 in *FT* chromatin in *clf* seedlings relative to Col determined by real-time quantitative PCR. The fold enrichments of *clf* over Col are shown, and the values shown are means±SD.

As noted above, PRC2 subunits repress but do not fully silence *FLC* and *FT* expression because both genes are still expressed at low levels in wild-type seedlings. It has been shown that active H3K4me3 is associated with *FLC* chromatin in *Arabidopsis* accessions which lack of *FRI* such as Col and Wassileskija (Ws) in which *FLC* expression is repressed [14], [64], and repressive H3K27me3 is also associated with *FLC* chromatin in these accessions in the absence of vernalization treatment [39], [64] (also see Figure 6A). However, it remains unknown whether *FLC* chromatin can simultaneously carry these two modifications as it is formally possible that these modifications could occur in two subpopulations of *FLC* chromatin and not in the same physical region of *FLC*. To examine whether *FLC* chromatin concomitantly carries both H3K4me3 and H3K27me3, we performed a sequential ChIP in which *FLC* chromatin from seedlings was immunoprecipitated first with anti-trimethyl H3K4 and second with anti-trimethyl H3K27. Both the region around TSS (*FLC-P2*) and 5′ part of Intron I of *FLC* (*FLC-I*) in part of the *FLC* chromatin concomitantly harbor H3K4me3 and H3K27me3 (Figure 7A). Similarly, using sequential ChIP we also found that the 5′ transcribed region (*FT-E*) and the middle of *FT* (*FT-I*) in part of the *FT* chromatin simultaneously harbor H3K4me3 and H3K27me3 (Figure 7A). In addition, we did not detect any DNA fragments from a heterochromatic locus *Ta3* [65] that lacks of H3K4me3 or from a constitutive expressed house-keeping gene *ACTIN 2* (*ACT2*) carrying abundant H3K4me3 (data not shown) but lacking of H3K27me3 (Figure 7A). Together, these data show that part of the *FLC* and *FT* chromatin simultaneously possesses the bivalent chromatin marks of active H3K4me3 and repressive H3K27me3.

We further investigated the interaction of H3K27 trimethylation with H3K4 trimethylation in *FLC* and *FT* chromatin. The H3K4 trimethylation state in these two loci was examined in *clf* seedlings by ChIP. Levels of trimethyl H3K4 in 5′ genomic *FLC* including *FLC-P1* and *FLC-P2* regions and in the 5′ transcribed region of *FT* (*FT-E*) and the middle of genomic *FT* (*FT-I*) were increased upon loss of CLF activities (Figure 7B and 7C), consistent with *FLC* and *FT* derepression in *clf*. Furthermore, the levels of trimethyl H3K4 in *MAF4* and *MAF5* were also increased in *clf* relative to Col (Figure 7B), in line with *MAF4* and *MAF5* derepression in *clf*. Together, these data suggest that the CLF-dependent H3K27 trimethylation suppresses H3K4 trimethylation in *FLC*, *MAF4*, *MAF5* and *FT*. Interestingly, the global levels of trimethyl H3K4 in *clf* were similar to those in Col (Figure 5C), indicating that CLF-containing PRC2-like complexes only suppresses the H3K4 trimethylation in their target-gene chromatin.

## Discussion

Our studies reveal that the *Arabidopsis* PRC2-like complex subunits CLF, EMF2 and FIE repress the expression of *FLC* and *FLC* relatives including *MAF4* and *MAF5*, and that CLF directly binds to and mediates the deposition of repressive H3K27me3 in these three loci. Furthermore, we show that during vegetative development *CLF* and *FIE* strongly repress *FT* expression, and that CLF directly interacts with and mediates the deposition of H3K27me3 in *FT* chromatin. Our results suggest that CLF-containing PRC2-like (CLF-PRC2) complexes containing EMF2 and FIE directly interact with and deposit into the *FLC*, *MAF4*, *MAF5* and *FT* chromatin repressive trimethyl H3K27 leading to the suppression of active H3K4me3 in these loci, and thus repress the expression of these flowering genes. Given the central roles of *FLC* and *FT* in flowering-time regulation in *Arabidopsis*, these findings suggest that CLF-PRC2 complexes play a significant role in control of the *Arabidopsis* flowering.

### PRC2 Subunits-Mediated Repression of *FLC* and *FLC* Relatives

Previous studies indicate that a PRC2-like complex containing VRN2, FIE and SWN or CLF might be involved in *FLC* repression in *Arabidopsis* plants grown in normal conditions [51]. In this study, we show that CLF is an essential component for *FLC* repression because CLF directly binds to *FLC* chromatin and loss of *CLF* function leads to a reduction in H3K27me3 and *FLC* derepression. SWN, a CLF relative, may also play a role in *FLC* repression because low levels of trimethyl H3K27 in *FLC* chromatin have still been detected in *clf* seedlings (Figure 6A) and simultaneous co-suppression of *SWN* and *CLF* leads to *FLC* derepression [51], though *swn* mutants do not display a phenotype [57]. In addition, we have found that EMF2, a CLF-interacting partner [57], represses *FLC* expression. Previously it has been shown that VRN2, an EMF2 relative, also interacts with CLF and represses *FLC* expression in the absence of vernalization [51], [66]. EMF2 and VRN2 can act in partial redundancy in PRC2-like complexes [57]; hence, these two proteins may act in partial redundancy to repress *FLC* expression. Furthermore, we have found that *CLF*, *EMF2* and *FIE* also repress the expression of *MAF4* and *MAF5*. Together, these findings suggest that these PRC2 subunits may form a CLF-PRC2 complex that directly represses *FLC*, *MAF4* and *MAF5* expression.

The *Drosophila* PRC2 complex contains four core components including E(z), Esc, Su(z)12 and p55, and these components are evolutionarily conserved in animals and plants (reviewed in [40], [41]). CLF and SWN, EMF2 and VRN, and FIE are homologs of E(z), Su(z)12, and Esc respectively. *Arabidopsis* has five homologs of p55 including MSI1 and FVE. MSI1 is part of a PRC2-like complex that regulates seed development [43], but is not involved in *FLC* repression [67]. *FVE*, a component in the autonomous pathway, represses *FLC* expression to promote flowering [5]. *fve* mutants grown under normal conditions, are phenotypically wild type except for late flowering [5], whereas *clf* mutants, *emf2* mutants and *FIE*-suppressed plants display pleiotropic phenotypes [47], [53], [55], suggesting that these three genes play a role in plant development that is much broader than that played by *FVE*. Interestingly, like *CLF*, *EMF2* and *FIE*, *FVE* also represses *MAF4* and *MAF5* expression (Figure S1). Together, these findings are consistent with a model in which a CLF-PRC2 complex composed of CLF, EMF2, VRN2, FIE and FVE selectively represses the expression of *FLC*, *MAF4* and *MAF5* to promote the floral transition in the absence of vernalization. In addition, SWN might also be part of this complex and may partially substitute for CLF. It is noteworthy that FVE can directly interact with a plant retinoblastoma protein (see the discussion below) [5], and future biochemical experiments are required to assess whether FVE is part of a CLF-PRC2 complex.

### A CLF-PRC2 Complex May Act in Concert with the Autonomous-Pathway Repressors to Repress *FLC* Expression in the Absence of Vernalizaition

The autonomous pathway includes six classic loci such as *FCA*, *FLD* and *FVE*, and these genes do not form a linear pathway [68]. This pathway is so named because mutations in these genes lead to late flowering in all photoperiods due to the elevated *FLC* expression (reviewed in [2]). FLD, a plant homolog of the human Lysine-Specific Demethylase 1 that has been found in histone deacetylase co-repressor complexes, is involved in the H3K4 demethylation (a mechanism associated with gene repression) and deacetylation of *FLC* chromatin [8], [17]. In addition, recent studies have shown that *FCA* functions closely with *FLD*, and that like *FLD*, it is involved in H3K4 demethylation of *FLC* chromatin [18]. In this study, we have found that removing *CLF* and *FCA* function leads to the synergistic *FLC* derepression, indicating that the CLF-PRC2 complex-mediated H3K27me3 acts in partial redundancy with the *FCA*- and *FLD*-mediated chromatin repression in *FLC* suppression in the absence of vernalization. In addition, our studies also suggest that the CLF-dependent H3K27 trimethylation may antagonize H3K4 trimethylation in *FLC* chromatin, indicating that H3K27 trimethylation may facilitate H3K4 demethylation in *FLC* chromatin. Furthermore, the *Drosophila* PRC2 complex has been shown to be associated with histone deacetylases, suggesting that histone deacetylation is also linked to the PRC2-mediated gene repression [69]. Interestingly, recent studies have shown that FVE can directly interact with a plant retinoblastoma protein of which the human homolog has been found to be associated with a histone deacetylase complex [70], and that *FVE* is indeed involved in the deacetylation of *FLC* chromatin [5]. Taken together, it is likely that a CLF-PRC2 complex may act in concert with the autonomous-pathway repressors such as FCA and FLD, and histone deacetylases to generate a repressive chromatin environment through histone deacetylation, H3K4 demethyaltion and H3K27 trimethylation, and thus represses *FLC* expression.

### Recruitment of PRC2 Subunits to the Target Loci

*FLC*, *FLM* and *MAF2-5* are close relatives and have similar genomic structures [3], [13]. Particularly, *MAF2*, *MAF3*, *MAF4* and *MAF5* are arrayed in a gene cluster (a tandem array) located at the bottom of Chromosome 5 [13]; however, *CLF* represses only *MAF4* and *MAF5*, but not *MAF2* or *MAF3* in this gene cluster. The CLF-dependent H3K27me3 occurs in *MAF4* and *MAF5*, but is absent from *MAF3* and *At5g65090* (the gene immediately downstream *MAF5*), suggesting that the H3K27 trimethylation in *MAF4* and *MAF5* is not the result of spreading from the neighboring genes. Furthermore, CLF specifically binds to *MAF4* and *MAF5*, but not to *MAF3* or *At5g65090*. This suggests that CLF is specifically recruited to the *MAF4* and *MAF5* loci, indicating that there are *cis*-regulatory DNA elements in these two genes that may function similarly to Polycomb-group response elements in *Drosophila* [40] to recruit a PRC2-like complex.

### PRC2 Subunits-Mediated *FT* Repression

PRC2 subunits CLF, EMF2 and FIE all strongly repress *FT* expression during vegetative development, suggesting that a PRC2-like complex containing CLF, EMF2 and FIE represses *FT* expression. To date, all known PRC2 complexes in animals and plants contain four core components including p55 or a p55 homolog (reviewed in [40], [41]); however, the p55 homolog directly involved in *FT* repression still remains elusive. FVE, a p55 homolog and an *FLC* repressor, is not directly involved in *FT* repression because *FT* is strongly repressed in *fve* due to the elevated *FLC* expression [67], indicating that the PRC2-like complex repressing *FT* expression might be different from the one involved in *FLC* repression. Consistent with this notion, we have found that H3K27 trimethylation in *FT* chromatin is nearly eliminated in *clf*, whereas low levels of trimethyl H3K27 in *FLC* chromatin have been detected in *clf*, indicating that CLF relatives such as SWN may partially substitute for CLF in the deposition of H3K27me3 in the *FLC* locus, but not in the *FT* locus.

Our studies suggest that the putative CLF-PRC2 complex directly deposits repressive H3K27me3 in *FT* chromatin to repress *FT* expression. *FT* chromatin can be simultaneously marked with active H3K4me3 and repressive H3K27me3; the CLF-dependent H3K27 trimethylation suppresses, but does not eliminate H3K4 trimethylation in *FT* chromatin (Figure 7A and 7C), consistent with that *FT* is repressed but not fully silenced by PRC2 subunits in vegetative development. Recent studies suggest that LHP1 specifically recognizes and binds to H3K27me3 deposited by PRC2-like complexes to maintain stable transcriptional gene repression [37], [38]. LHP1 has been shown to directly bind to the *FT* locus and loss of LHP1 activities leads to *FT* derepression and early flowering [36], [37]. Hence, the CLF-dependent H3K27me3 in *FT* chromatin may be ‘read’ by LHP1 resulting in stable *FT* repression during vegetative development.

### Possible Role of the CLF-PRC2 Complex-Mediated *FT* Repression in the Regulation of *FT* by Photoperiod

The PRC2-mediated transcriptional gene repressing mechanisms are conserved in animals and plants (reviewed in [40], [41]). Our studies suggest that during vegetative development, *Arabidopsis* exploits these evolutionarily conserved ancient gene-repressing mechanisms to control *FT* expression; specifically, a CLF-PRC2 complex is utilized to repress, but not to fully silence *FT* expression in vegetative development. In the absence of PRC2 subunits, *FT* is highly activated; for instance, levels of *FT* transcripts in *clf* seedlings are about 200 fold of those in the wild type. It has been shown that in the wild type *FT* is expressed in vasculature such as veins of leaves where day length is perceived (reviewed in [1]). Previous studies show that loss of CLF activities leads to a strong derepression of *AG* throughout the leaf including veins and mesophyll cells [47]; hence, loss of CLF-PRC2-complex activities may well lead to *FT* derepression throughout the leaf including veins. Overexpressing *FT* via a strong constitutive viral promoter (35S) has been shown to give rise to extremely early flowering independent of the photoperiods [29], [30]. Thus, it is critical for plants to keep *FT* to be expressed at low levels for preventing precocious flowering and for the regulation of *FT* by the photoperiods. PRC2 subunits, likely functioning in the context of a CLF-PRC2 complex, maintain *FT* expression at basal lower levels in vegetative development, which may serve to provide some room for the elevated *FT* expression in response to photoperiods and thus enable the photoperiodic control of flowering time in plants.

## Materials and Methods

### Plant materials and growth conditions

*Arabidopsis thaliana clf-81* [48], *fca-9* [7], *fve-4* [4], *emf2-1* [52], [53] and *FIE*-suppressed plants derived from a homozygous transgenic line [55] were described previously. Plants were grown under cool white fluorescent light in long days (16 h light /8 h night) at about 22°C.

### RNA isolation, reverse transcription and quantitative PCR assays

Total RNAs from aerial parts of 7 to 10 day-old seedlings grown in long days were extracted as described previously [17]. cDNAs were reverse-transcribed from total RNAs with Moloney murine leukemia virus reverse transcriptase (Promega).

Real-time quantitative PCR was performed on an ABI Prism 7900HT sequence detection system using SYBR Green PCR master mix (Applied Biosystems) as described previously [17]. Each sample was quantified at least in triplicate and normalized using *TUB2* (*At_5g62690*) as the endogenous control. Primers used are specified in Table S1.

### Histone extraction and immunoblotting

Histone protein extraction and Western analysis were performed as described previously [18], [71]. Briefly, total histones were extracted from about 10-day-old seedlings, separated in an SDS-PAGE gel, and subsequently were transferred to a 0.2-µm nitrocellulose membrane (Bio-Rad). The protein blots were first probed with anti-trimethyl H3K27, anti-dimethyl H3K27 (Millipore) and anti-trimethyl H3K4 (Abcam), and followed by anti-H3 (Millipore). The chemiluminescent SuperSignal West Pico system (Pierce) was used to develop the protein blots according the manufacturer's instructions.

### Chromatin immunoprecipitation (ChIP)

The ChIP experiments were performed as described previously [65] using seedlings. Rabbit polyclonal anti-trimethyl-histone H3 (Lys 4) (Abcam), anti-trimethyl-histone H3 (Lys 27) (Upstate) and anti-GFP (Invitrogen) were used in immunoprecipitation experiments. Amounts of the immunoprecipitated genomic DNA were examined by PCR or quantified by real-time quantitative PCR. The PCR amplification of a genomic region was usually tried at several cycle numbers to identify a cycle number at which the amplification of DNA fragments in the immunoprecipitated DNA samples did not reach the plateau phase. Quantitative measurements of various regions of *FLC*, *MAF4*, *MAF5* and *FT* were performed using SYBR Green PCR master mix (Applied Biosystems). Primers used to amplify *FLC-P1*, *FLC-P2*, *ACTIN 2*, *TUB2* and *TUB8* were described previously [17], [72], and other primers used are specified in Table S1. Each of the immunoprecipitations was repeated independently once, and each sample was quantified in triplicate.

### Sequential ChIP analysis

The sequential ChIP experiments were performed as previously described [73] with modifications. Briefly, chromatin from Ws seedlings was immunoprecipitated with anti-trimethyl H3K4, subsequently eluted in a solution of 500 mM NaCl, 30 mM DTT and 0.1% SDS at 37°C, and was further diluted in a lysis buffer [65] supplemented with 1× Roche protease inhibitor cocktails (-EDTA). The eluted chromatin was subsequently immunoprecipitated with anti-trimethyl H3K27; DNA fragments were recovered and purified for PCR analysis.

## Supporting Information

Figure S1 *FVE* represses *MAF4* and *MAF5* expression. Total RNAs were extracted from Col, *fve* and *fca* seedlings grown in long days. *MAF4* and *MAF5* were de-repressed in *fve*, but not in *fca*.
(4.35 MB TIF)

Table S1 (0.03 MB DOC)
