# Supplementary figures and images for: Repression of FLOWERING LOCUS C and FLOWERING LOCUS T by the Arabidopsis Polycomb Repressive Complex 2 Components
Source: PLoS One. 2008 Oct 14;3(10):e3404. doi: 10.1371/journal.pone.0003404 (PMC2561057; doi:10.1371/journal.pone.0003404)

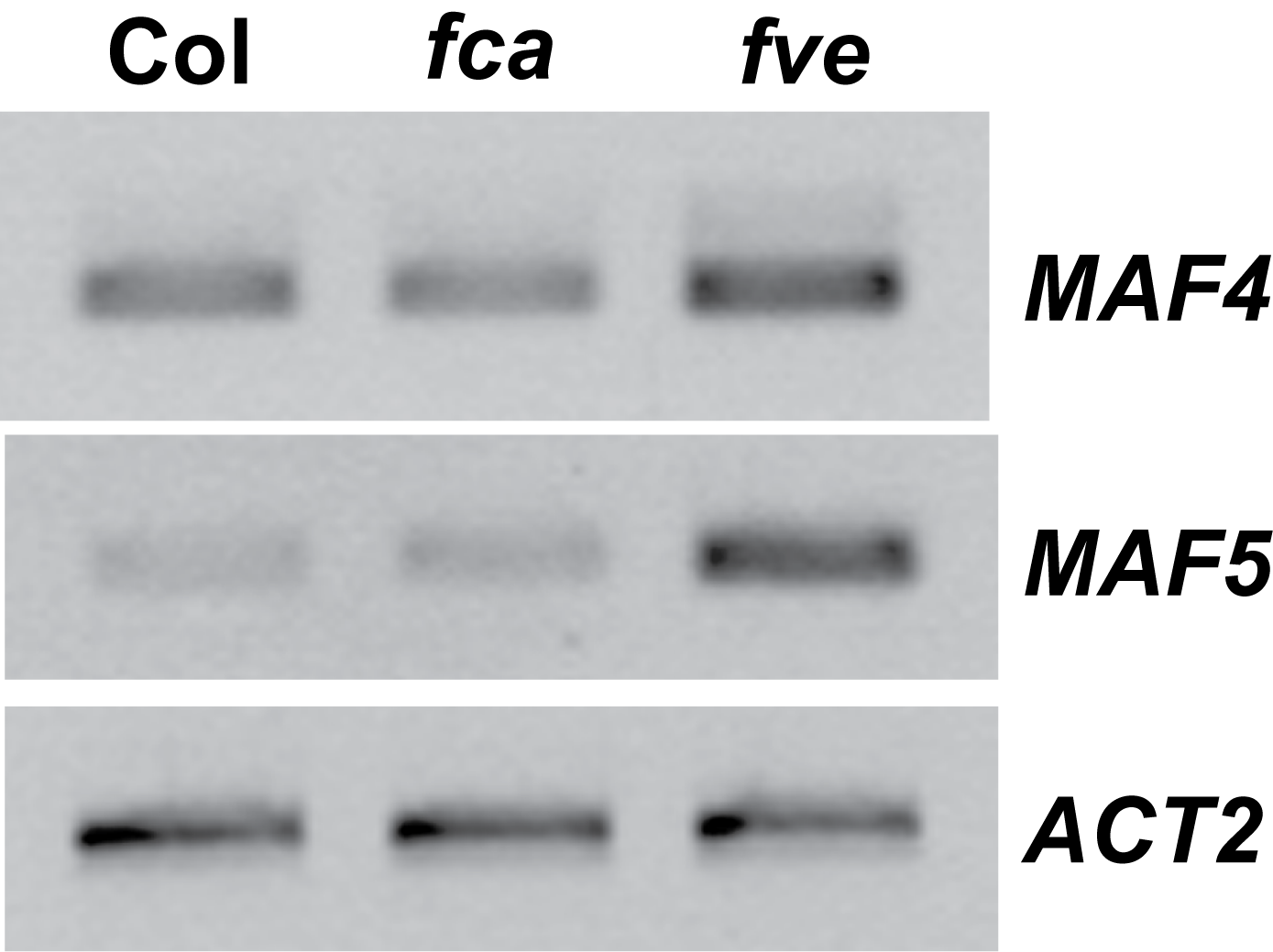

Supplement: Figure S1 — FVE represses MAF4 and MAF5 expression. Total RNAs were extracted from Col, fve and fca seedlings grown in long days. MAF4 and MAF5 were de-repressed in fve, but not in fca. (4.35 MB TIF) [file pone.0003404.s001.tif]
