# Supplementary material for: Repression of FLOWERING LOCUS C and FLOWERING LOCUS T by the Arabidopsis Polycomb Repressive Complex 2 Components
Source: PLoS One. 2008 Oct 14;3(10):e3404. doi: 10.1371/journal.pone.0003404 (PMC2561057; doi:10.1371/journal.pone.0003404)
Supplement: Table S1 — (0.03 MB DOC) [file pone.0003404.s002.doc]

**Table S1.** Sequences of primers used in experiments of RT-PCR and ChIP-PCR.

Experiments Amplified regions Sequences

**RT-PCR** *FLC* Forward: CCGAACTCATGTTGAAGCTTGTTGAG

Reverse: CGGAGATTTGTCCAGCAGGTG

*MAF2* Forward: AACTCGGAATTATCTGCCACTCAAAG

Reverse: CTTCCCCCATCATTAGTTCTGTCTTC

*MAF3* Forward: GAAAGGGAGAAGTTGCTGATAGAAGAG

Reverse: AGCACAAGAACTCTGATATTTGTCTAC

*MAF4* Forward: GCTTCTCCTCAGGTGATAGCATG

Reverse: CTGCTCTTCCAGGGACTTTAGAC

*MAF5* Forward: TGTGTCGGAAGAGTGAAGCCAT

Reverse: CTGATGATCTTGGCCATGCTGT

*FT* Forward: CCTCAGGAACTTCTATACTTTGGTTATGG

Reverse: CTGTTTGCCTGCCAAGCTGTC

**ChIP-PCR** *FLC-P0* Forward: TGGATTGATGTGGGGCACTATTAAGT

Reverse: GGTTGTTCCCTCCAAACCAATTTGAG

*FLC-I* Forward: GTCATTCACGATTTGTTTGATACGATCTG

Reverse: GATCTCCCGTAAGTGCATTGCA

*MAF4-I* Forward: GCTAGTTTCTTGGTAGCTCGGCTG

Reverse: CATTCTTACTTCGTGTCGTCTGTGATC

*MAF5-I* Forward: CGTGGTGGTAATCCGTAATTCATGT

Reverse: CAAATGGCACTCGTTTCCACTAGA

*MAF3-I* Forward: CTGTGTGAATAGAGCCTATGCGTTACC

Reverse: CTTGAACAGCATTGAGAATGTATCAACACG *FLM* Forward: TGGTGATTCGTTCTTGATGTTGTTTAAG

Reverse: GTTACCACTAACCCCAAATAATCTCTCAAC

*FT-E* Forward: GAGACCCTCTTATAGTAAGCAGAGTTG

Reverse: GGGAGTTCAAGTGAAAGAACCAAAGT

*FT-I* Forward: CCAGATGTTCCAAGTCCTAGCAACC

Reverse: GGTGTGGGCTTTTTTGGGAGAC

*At5g65090* Forward: GTGGTAGTGAAGAATCAAGAGCACC

Reverse: GAACCCTAAAGTTCTCAGGCTCCAC
